# Advertising Sheet

**Published:** 1861-08

**Authors:** 


### ADVERTISING SHEET.

We have taken pains to select such business notices only as are of interest and importance to physicians. The Book Merchants whose advertisements appear, have on hand the most recent publications in Medicine, Surgery and the Collateral Sciences, and we understand are offering great inducements to the physicians who buy books.

The Druggists whose notices are also to be found in the advertising sheet, are men to be depended upon as dealing only in the most carefully selected goods to be found in market, while Prescriptions will be dispensed by them in the best manner. We have also had opportunity to know, and have carefully examined the stock of Spirits and Wines offered at wholesale by Mr. Ayers, and are satisfied of the purity and superiority of this stock. These notices are all designed quite as much for the benefit of those who buy, as those who sell, and though we receive pay for advertisements, yet we do not receive *all advertisements*, even for the best of pay.

